# Neuroimaging Links Between Heart Failure and Depression—A Narrative Review

**DOI:** 10.3390/brainsci14121283

**Published:** 2024-12-20

**Authors:** Giacomo Deste, Carlo Lombardi, Roberto Gasparotti, Antonio Vita, Daniele Corbo

**Affiliations:** 1 Department of Clinical and Experimental Sciences, University of Brescia, 25123 Brescia, Italy; antonio.vita@unibs.it; 2 Department of Mental Health and Addiction Services, ASST Valcamonica, 25040 Esine, Italy; 3 Institute of Cardiology, Department of Medical and Surgical Specialties, Radiological Sciences and Public Health, University of Brescia, 25123 Brescia, Italy; 4 Cardiology Unit, ASST Cremona, 26100 Cremona, Italy; 5 Department of Medical and Surgical Specialties, Radiological Sciences and Public Health, University of Brescia, 25123 Brescia, Italy; 6 Neuroradiology Unit, ASST Spedali Civili of Brescia, 25123 Brescia, Italy; 7 Department of Mental Health and Addiction Services, ASST Spedali Civili of Brescia, 25123 Brescia, Italy

**Keywords:** depression, neuroimaging, MRI, heart failure, cognition

## Abstract

Background and objective: It is commonly known that there is a connection between heart disease and depression symptoms. Compared to heart failure patients without concurrent depression, those with depressive symptoms are more likely to have longer hospital stays and more outpatient visits following discharge. Although the exact neurobiological mechanisms causing the correlation between heart disease and depression symptoms are unknown, it is thought that vascular abnormalities may be a major factor. The purpose of this review was to examine the connection between brain networks linked to depression and heart failure (HF). Methods: PRISMA guidelines were followed. We included studies that reported both heart failure as well as depression and neuroimaging. Results: We identified 159 papers, but only 12 articles were included. Our findings show that reduced cerebral blood flow (CBF) following HF, along with other contributing factors such as chronic inflammation and neurovascular dysfunction, can lead to significant brain tissue damage and disruption of neural networks. The resulting alteration in the brain increases the risk of developing depression, as the neural circuits responsible for emotional regulation become compromised. Conclusions: Individuals with heart failure (HF) exhibit reduced regional cerebral blood flow across multiple brain areas, many of which are critical for mood regulation and are commonly implicated in depression, such as the left frontal cortex and right hippocampus.

## 1. Introduction

Heart failure (HF) is a significant public health concern, responsible for approximately one million hospital admissions annually due to the negative health effects associated with cardiac dysfunction [1]. It is well known that aging and vascular processes in HF damage the brain, including altered cerebral hemodynamics [2,3]. HF patients demonstrate a significant reduction in resting cerebral blood flow (CBF), which is approximately 31% lower compared to healthy individuals of the same age. This marked decrease suggests impaired cerebral perfusion likely stemming from reduced cardiac output and altered vascular dynamics associated with HF [4,5].

Reduced cerebral blood flow can lead to oxygen deprivation and nutrient shortages in critical brain regions, contributing to cognitive deficits, emotional dysregulation, and neurological symptoms frequently observed in HF patients [6,7]. Studies have demonstrated that brain regions particularly sensitive to reduced oxygen supply, including the frontal lobes and parahippocampal areas, are frequently impacted in conditions of cerebral hypoperfusion [8]. Reduced oxygenation in these regions can disrupt neural connectivity and lead to structural changes, such as white matter hyperintensities or neuronal atrophy [9], further exacerbating impairments in key cognitive processes, such as attention, executive functioning, and decision-making. Additionally, evidence from numerous studies highlights ischemic-related brain changes in HF patients, such as decreased axonal integrity and white matter hyperintensities [10,11].

While mood disorders are equally prevalent among patients with heart failure (HF), they have received significantly less research attention compared to neurological aspects of the condition, such as cognitive deficits and vascular dementia. Between 25% and 75% of HF patients show impairments on neuropsychological tests, reporting issues such as memory decline and difficulties in cognitive capacities [11]. Most significantly, HF affects various cognitive functions, particularly different forms of memory, attention, and executive skills like concentration and problem-solving [12,13]. Moreover, depression, anxiety, and other psychological issues often accompany HF, but executive skills like concentration and problem-solving are also compromised, profoundly affecting patients’ quality of life, treatment adherence, and clinical outcomes [14]. Despite their widespread impact, these mental health concerns remain underexplored, with limited studies addressing their mechanisms, progression, or optimal interventions. By contrast, the neurological consequences of HF, such as cognitive impairments and cerebral hypoperfusion, have been more extensively studied. Based on clinical diagnoses and depression scales, an estimated 21% to 36% of HF patients experience depression, with as many as 42% demonstrating clinically significant depressive symptoms [14,15,16]. Depression in HF patients is associated with an increased risk of hospitalization, cardiac events, and mortality [17,18].

There is growing evidence that cerebral hypoperfusion may contribute to both cognitive impairment and depression in HF patients. Neuroimaging studies reveal pathological changes in the brains of individuals with HF, including increased atrophy and white matter hyperintensities (WMHs), especially in the frontal subcortical circuits [15]. One of the key features observed in neuroimaging studies of HF patients is reduced cerebral blood flow, especially in areas that are involved in mood regulation, such as the prefrontal cortex and the limbic system. fMRI studies have shown that HF patients exhibit decreased activation in brain regions involved in emotional processing and regulation, such as the prefrontal cortex and amygdala [8]. These alterations are associated with elevated depressive symptoms, as observed in healthy older adults and other patient groups [19,20].

Although depression is likely linked to HF through shared pathological pathways, such as inflammation, cerebral hypoperfusion, and neuroendocrine dysregulation, relatively few studies have specifically examined the neural mechanisms underlying this connection. The overlap in the affected brain regions, such as the prefrontal cortex and hippocampus, suggests a common neurobiological basis for these conditions. However, the specific neural mechanisms and brain alterations that underpin depressive symptoms in HF patients are not yet fully understood. The aim of this narrative review was to provide a detailed overview of the existing evidence on how structural, functional, or metabolic brain alterations observed through neuroimaging modalities, such as MRI, fMRI, or PET, contribute to the development or progression of depressive symptoms in individuals with HF. By synthesizing findings from diverse studies, this review seeks to highlight shared mechanisms of depression and HF, identify gaps in current knowledge, and propose directions for future research.

## 2. Materials and Methods

### 2.1. Study Experimental Design

This narrative review was conducted following a re-adaptation of the PRISMA flow [21]. The research was carried out in the period between August 2024 and October 2024.

#### 2.1.1. Inclusion Criteria

As previously stated, the aim of this narrative review was to incorporate all research studies that met the following criteria: neuroimaging studies exploring the link between HF and depression. To be included, research studies had to be written in English, present in full text, and provide sufficient methodological details. There were no restrictions based on the age of participants or the publication year of the studies. Depression had to be assessed using a validated measure, such as the Structured Clinical Interview (SCID-I) based on DSM-IV criteria conducted by trained interviewers or the Hamilton Depression Scale. Samples consisting of animal samples were excluded.

#### 2.1.2. Study Selection

**Figure 1:**
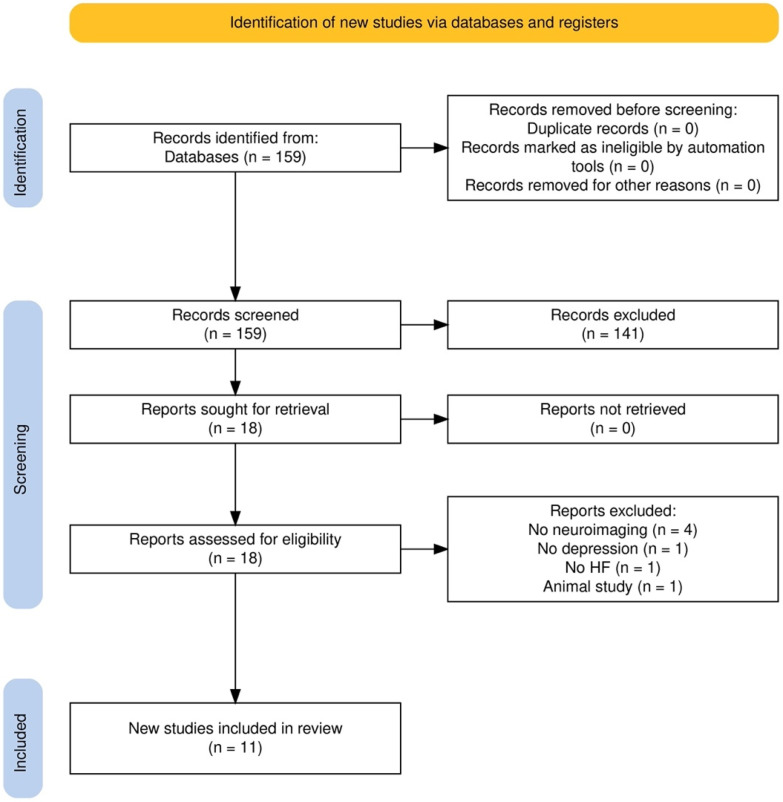
Flowchart of the narrative review process.

To assess the relevant literature in this field, this qualitative review included works published up to October 2024, identified through a search of open-access databases such as PubMed, Google Scholar, Web of Science (WoS), and Scopus. The initial search in all databases utilized a combination of the following terms: [(heart failure) AND (depression) AND ((fMRI) OR (neuroimaging))] without specifying a certain period of publication dates. References from the identified articles, additional sources, and relevant literature reviews were also reviewed for potential inclusion in the analysis. The processes of database selection, exclusion criteria application, secondary searches, and final article selection—outlined in the flow diagram (Figure 1)—were carried out by one of the authors (D.C.).

Our initial search yielded 159 results. After searching for any duplicates, 159 unique articles remained and were screened based on their titles and abstracts. Of these, 141 were excluded because they did not cover all topics of interest (HF, depression and neuroimaging). The full texts of the remaining 18 articles were then evaluated for eligibility, and their reference lists were reviewed for additional relevant studies, though this process did not identify any new articles. Subsequently, the full texts of the final 18 articles were reassessed to ensure they adhered to the inclusion and exclusion criteria, resulting in the exclusion of 7 articles (four because they did not address neuroimaging, one because it did not address depression, one because it did not address HF, and one because it was an animal study). Ultimately, 11 articles met the inclusion criteria.

From each included study, the following information was extracted: (1) authors and year of publication; (2) sample size; (3) participant characteristics, including demographic details and clinical diagnosis; (4) neuroimaging study features, such as imaging type and outcome measures; and (5) main findings.

## 3. Results

All results are summarized in Table 1.

Although the number of studies found is small, the neuroimaging methodologies used are varied and allow for different inferences. Most studies find depression-related outcomes without having a specific hypothesis, while some distinguish groups of depressed subjects to test for differences with controls and HF subjects.

### 3.1. Altered Cerebral Hemodynamics

Almeida et al. [19] examined the frequency and intensity of white matter hyperintensities from MRI in patients with heart failure without depression, patients with late-life depression with heart failure, and healthy elderly volunteers using a visual rating scale. They concentrated their research on the frontal lobe since it has been suggested that this is the area of the brain where white matter hyperintensities are preferentially seen in people with late-life depression [19]. The severity of frontal periventricular white matter hyperintensity and depression scores on the Hamilton scale were significantly correlated in the heart failure and depression group, despite the fact that there were no significant group differences in frontal region white matter hyperintensities. No other parts of the brain showed any significant results. These findings strengthen early evidence that the severity of depressive symptoms in heart disease may be influenced by white matter hyperintensities, particularly in frontal regions. In the Alosco et al. study [26], 100 HF patients had their global cerebral blood flow velocity (CBF-V) measured by transcranial Doppler ultrasonography. They also took the Beck Depression Inventory-II to measure depressive symptomatology and a battery of cognitive tests to evaluate their global cognition, attention/executive function, and memory skills. These operations were carried out at baseline and at a follow-up after 12 months. CBF-V decreased during the course of the 12-month period, according to repeated measurements. Even after adjusting for baseline covariates, medical conditions, and demographic characteristics, regression models revealed that lower baseline CBF-V predicted worse attention/executive function performances, a reduction in working memory performance, and increased depressed symptomatology at the 12-month follow-up. At a 1-year follow-up in HF, cerebral perfusion decreased with time and was linked to worse cognitive performance and more depressed symptoms. Sabayan et al. [30] looked at the relationship between cardiac hemodynamics and brain aging characteristics in elderly individuals who lived in the community. They examined the relationship between brain features and MRI measures of cardiac hemodynamics, such as left ventricular stroke volume (LVSV) and cardiac output (CO), using data from a sub-study. Poorer executive function and processing speed performance were linked to lower LVSV and CO. Features of brain alterations are linked to a progressive decline in heart performance. Cardiac, cognitive, and mood disorders often coexist in older adults presenting with symptoms related to the heart or brain.

### 3.2. Structural Alterations

Woo et al. [22] used high-resolution T1-weighted magnetic resonance imaging to study HF patients with some depression symptoms and healthy control participants in order to measure regional hippocampus volume loss. They also used morphometric techniques to evaluate localized surface alterations. Surface morphometry was used to measure regional differences, and two-sample t-tests were used to evaluate the volume differences of the hippocampus across groups. Compared to controls, HF patients had decreased hippocampus sizes. Volume reductions were primarily observed in the mid-to-posterior Cornu Ammonis (CA)3 region, which receives input from the dentate gyrus terminating in the stratum lucidum and is regarded as the hippocampus’s pacemaker. Additional volume decreases were noted in the subiculum and CA1, a region critical for various learning and memory functions. In HF, the hippocampus exhibits a regional decrease in volume, which might be a factor in the condition’s associated depression and short-term memory loss [32,33]. A 24-month longitudinal study of individuals with systolic heart failure (HF), ischemic heart disease (IHD), and controls was conducted by Almeida et al. [25] in MRI. They examined how the amount of gray matter in the brain changed over time, evaluating changes in mood and cognitive performance after two years. While the three research groups’ changes in overall gray matter volume and cognitive performance were comparable, HF individuals had signs of worsening anxiety and depression symptoms. In comparison to controls and, to a lesser extent, individuals with IHD, HF was linked to a mild regional loss of gray matter in the right and left thalamus, left caudate, left and right posterior cingulate, left and right parahippocampal gyri, left superior and middle temporal gyri, and right inferior parietal lobule. When compared to controls who are cardiologically healthy, HF and IHD are not linked to a disproportionate loss of cerebral gray matter or cognitive deterioration over a two-year period. Over a two-year period, adults with heart failure (HF) exhibited significantly higher levels of anxiety and depressive symptoms compared to healthy controls. This pronounced difference in emotional well-being is closely associated with a relative reduction in gray matter volume within brain regions integral to emotional regulation, such as the prefrontal cortex and limbic system. These structural changes in the brain may impair the ability to manage stress and regulate mood effectively, potentially exacerbating psychological distress in HF patients. The findings underscore the critical link between neuroanatomical alterations and the heightened prevalence of emotional symptoms in individuals with heart failure. Woo et al. [27] aimed to evaluate anterior insular metabolites in order to identify the mechanisms behind autonomic, pain, and neuropsychologic disturbances in heart failure. They focused on the major metabolites, assigning peaks for NAA at 2.02 ppm, Cr at 3.02 ppm, Cho at 3.2 ppm, and MI at 3.56 ppm, in the bilateral anterior insulae of HF and controls. They calculated peak areas and metabolites expressed as ratios, such as NAA/Cr, Cho/Cr, and MI/Cr. Compared to controls, HF patients had substantially higher Cho/Cr ratios on the left anterior insula, which indicated glial growth or damage, and lower NAA/Cr levels on the right anterior insula, which suggested neuronal loss or dysfunction. The groups’ MI/Cr ratios did not differ from one another. The altered autonomic, pain, and neuropsychologic processes observed in HF may be caused by a loss of right anterior insular neurons and increase in the number of glial cells on the left anterior insula. The anterior insular cortex plays a key role in regulating mood and anxiety [27], and abnormalities in its metabolites may contribute to the onset of depression. Woo et al. [28] assessed the degree of brain damage in HF using magnetic resonance T2 relaxometry. T2-weighted and proton-density images were obtained from 49 controls and 13 HF patients. Sites that regulate autonomic, analgesic, emotional, and cognitive functions (hypothalamus, raphé magnus, cerebellar cortex, deep nuclei, and vermis; temporal, parietal, prefrontal, occipital, insular, cingulate, and ventral frontal cortices; corpus callosum; anterior thalamus; caudate nuclei; anterior fornix and hippocampus) showed higher T2 relaxation values, indicating that these areas of the brain were injured. T2 levels in control vs. HF individuals were not greater in any part of the brain. In HF patients, structural damage to the brain surfaced in regions related to autonomic, pain, emotion, language, and cognitive function. Pan et al. [29] used brain MRI to visually inspect the hippocampus for global and regional tissue alterations in HF and control patients, as well as the mammillary bodies and frontal cortex for global tissue abnormalities. The left frontal brain, right hippocampus, and right mammillary body all showed notable global alterations. Cortical abnormalities in the right hippocampus and left frontal cortex, but not in the mammary body, are confirmed by comparing the visual approach with specialist MRI techniques. Visual inspection of brain MRI can identify damage in HF in regions such as the left frontal cortex and right hippocampus that control depression and executive function [29]. Evaluation of damage to these structures and the effects of possible therapies for this damage may be made easier with the use of visual MRI assessment in HF. The Modified Mini Mental State assessment (3MS) and brain MRI were conducted on sixty-nine heart failure patients in Alosco et al.’s research [31]. The 2 min step test (2MST), a quick assessment of physical fitness, was completed by every participant. They studied the relationships between three measures of global brain integrity—whole brain cortical thickness, total cortical gray matter volume, and total white matter volume—and cognitive function and physical fitness. The findings showed that a thinner cortex and a smaller gray matter volume were linked to worse performance on the 2MST. Subsequent studies revealed that lower 3MS scores were linked to decreased cortical thickness and gray matter volume. Reduced structural brain integrity is linked to poor physical fitness, which is prevalent in HF patients.

### 3.3. Functional Alterations

In the study of Bremner et al. [23], patients with and without significant depression who had coronary artery disease (CAD) underwent high-resolution PET brain imaging and SPECT and [Tc-99m] sestamibi heart imaging under control and mental stress circumstances. Compared to CAD patients without depression, those with CAD and significant depression exhibited a relative failure of medial prefrontal/anterior cingulate activation following mental stress, as well as greater activation of the parietal cortex. However, rostral regions of the anterior cingulate cortex were more activated in depressed CAD patients with stress-induced myocardial ischemia than in those without. These results support the idea that the mechanism of elevated risk for CAD morbidity and death in CAD patients diagnosed with significant depression involves brain regions linked to stress and depression. Ichijo et al. [24] used near-infrared spectroscopy (NIRS) to quantify and compare frontal brain activity in HF patients and control participants during a verbal fluency task. According to NIRS, the HF group’s frontal brain activity was noticeably less than that of the control group. They then looked at the relationships between neuropsychological tests and frontal brain activity. Frontal brain activity was significantly correlated with verbal fluency tasks, the Mini-Mental State Examination, and the State-Trait Anxiety Inventory, but not with the Center for Epidemiologic Studies Depression Scale. Patients with HF had lower frontal brain activity as measured by NIRS, which is linked to impaired cognitive performance and a high anxiety level.

## 4. Discussion

In this review, we wanted to explore the links between HF and depression and understand the effects from a neuroimaging point of view. Although there were not many articles investigating this aspect, we certainly delved into the brain mechanisms that link the development of depression to HF. Depression and heart failure seem to share multiple pathogenic pathways, including heightened platelet reactivity, chronic inflammation, dysregulation of the neuroendocrine system, the presence of arrhythmias, and the adoption of high-risk behaviors [34]. These overlapping mechanisms provide a biological foundation for the shared neural correlates observed in both conditions. For instance, inflammation and neuroendocrine dysregulation may contribute to structural and functional brain changes, such as reduced gray matter volume or altered connectivity in regions involved in mood regulation and autonomic control. Similarly, platelet reactivity and arrhythmias could impair cerebral perfusion, leading to hypoxia-related brain damage that exacerbates both cognitive and emotional symptoms. The structural and functional changes in the brain observed after HF may act as significant predisposing factors for the onset of depression. In heart failure, cerebral hypoperfusion is common, and lower CBF was associated with worse memory and attention/executive function [26]. It was also discovered that, in individuals with HF, lower cerebral blood flow was a predictor of higher depressive symptomatology at a 1-year follow-up [26]. More specifically, it was discovered that the primary cause of higher levels of depression symptoms a year later was the middle cerebral artery’s lower blood flow velocity [35]. These results provide evidence to the theory that the negative effects of cerebral hypoperfusion on the brain are at least partially responsible for depression in older persons with HF. Additionally, it has been shown that, in HF patients, altered autonomic, mood, cognitive, and language and speech regulatory sites may be linked to decreased CBF [10,27,36]. Multiple brain locations, including the vascular beds over the frontal, parietal, and occipital cortices as well as the hippocampus, thalamus, and cerebellar sections, exhibit regional CBF decrease in individuals with HF [24,25]. It has been observed in some MRI experiments that most of these brain locations also exhibit brain tissue damage. Brain structural abnormalities brought on by HF are linked to cognitive decline and depressive symptoms [27]. Decreased CBF in these areas probably causes tissue alterations, which may alter the severity of depression symptoms in HF patients. It has also been shown that heart failure patients had smaller global hippocampus sizes than control participants due to the same mechanisms. These volume losses were concentrated in particular locations, primarily CA1 and, to a lesser degree, the subiculum. Depression and other mood disorders in HF are probably exacerbated by the damage to the hippocampus and the hippocampus–mammillary body circuitry [22]. In one study, HF patients showed decreased executive function (frontal cortex) and more hippocampal depression symptoms than control participants [29]. It has been proposed that even the assessment, also visual, of the hippocampus and frontal cortex could become a method, in HF patients, to evaluate the onset of depression and to manage it better. White matter hyperintensity was shown to be significantly correlated with the intensity of depression symptoms in the frontal area of depressed heart failure patients [19]. This supports the theory that vascular injury to white matter tracts linking brain regions important for mood regulation may be the cause of the onset of depressive symptoms in conjunction with HF. Additionally, compared to HF patients without depression, it has been observed that the frontal cortex of depressed HF sufferers is less activated during stress activities [23]. Reduced activation of the frontal cortex in these patients aligns with previous research on depression, suggesting that dysfunction in this brain region may significantly contribute to the heightened risk of cardiovascular events observed in individuals with depression [20]. The frontal cortex is crucial for regulating stress responses, decision-making, and emotional processing. These findings emphasize the intricate connection between brain function and heart health, highlighting the potential role of frontal cortex dysfunction as a possible predisposing mechanism of depression following HF. It is possible that the hypoperfusion-induced atrophy might also result in less brain activity. It is interesting to note that HF patients who were depressed also showed higher parietal cortex activity, which may be a neural correlate of hypervigilance or heightened awareness in reaction to stress [24]. According to this research, these patients should adopt coping mechanisms that lessen stress and anxiety, useful in the prevention of morbidity and mortality in some vulnerable patients. It has also been seen that, for these patients, the reduction in physical activity is associated with decreased gray matter volume and cortical thickness [26], suggesting that another strategy to improve their condition is to increase physical activity. This review, to the best of our knowledge, is the only one to investigate brain alterations due to HF and depression, although it has the limitation that the number of studies found is relatively small. Future studies need to understand the mechanisms underlying this deterioration in brain tissue following HF and identify and develop interventions that may prevent the onset of depression. According to our findings, early diagnosis and management may be facilitated by knowledge of the neuroimaging correlations between depression and cognitive impairment in HF patients. For example, routine brain imaging may become a useful tool in identifying at-risk patients who may benefit from early cognitive and psychological interventions. The identification of brain regions affected by depression in HF opens up the potential for targeted therapies. Neuroprotective treatments (medication, exercise, brain exercise) that aim to improve cerebral perfusion and restore neurochemical balance could be beneficial in addressing mood in HF patients.

## 5. Conclusions

Our results show the existence of brain alterations following HF that may favor the development of depression. One of the key features observed in neuroimaging studies of HF patients is reduced cerebral blood flow, especially in areas that are involved in mood regulation, such as the prefrontal cortex and the limbic system. fMRI studies have shown that HF patients exhibit decreased activation in brain regions involved in emotional processing and regulation, such as the prefrontal cortex and amygdala. Furthermore, hippocampal damage, primarily affecting the CA1 subregion, suggests a neuroanatomical basis for the mood disturbances frequently observed in HF patients. Depressed HF patients also show increased activation in the rostral anterior cingulate cortex, linking this region to the emotional challenges seen in this population.

To build upon these findings, further longitudinal neuroimaging studies are needed, particularly those focusing on activation patterns using fMRI. Such studies should involve larger and more diverse populations, with more rigorous inclusion and application criteria. These efforts are crucial for confirming existing results and identifying effective neuroprotective strategies that could prevent the development of depression in individuals with HF, ultimately improving patient outcomes and quality of life.

## Figures and Tables

**Table 1:** Neuroimaging studies: research articles investigating neural structural plasticity changes due to the effect of depression and heart failure (MDD: major depressive disorder; HC: health control; HFnD: heart failure not depressed; HFD: depressed heart failure; CAD_D: depressed coronary artery disease; CAD_nD: coronary artery disease not depressed; IHD: ischemic heart disease).

Authors	Year	Diagnosis	Study Sample (N)	Age (Mean)	Sex at Birth (% Female)	Type of Imaging	Outcome Measures	Main Results
[19]	2005	cardiac disease; depression	14 HC; 10 HFnD; 8HFD	HC: 73.4; HFnD: 74.2; HFD: 75.6	HC: 71%; HFnD: 20%; HFD: 62%;	MRI	Frequency and severity of white matter hyperintensities	Correlation between the frontal periventricular white matter hyperintensity and depression
[22]	2015	heart failure	HF: 17; HC: 34	HF: 54.4; HC: 52.3	HF: 29%; HC: 29%	MRI	Regional hippocampal volume loss	Regional volume reduction in HF
[23]	2019	coronary artery disease; depression	CAD_D: 17; CAD_nD: 21	CAD_D: 58; CAD_nD: 61	CAD_D: 19%; CAD_nD: 29%	PET-SPECT	Effect of stress on brain in CAD_D patients	Increased activation in rostral portions of the anterior cingulate in CAD_D
[24]	2020	heart failure; depression	HF: 35; HC: 28	HF: 70.5; HC: 70.6	HF: 22.4%; HC: 40%	rs-MRI	Associations between frontal brain activity and depressive symptoms in HF	Frontal brain activity assessed was reduced
[25]	2013	heart failure; depression	HF: 19; IHD: 45; HC: 45	HF: 67.7; IHD: 66.3; HC: 69.1	HF: 26%; IHD: 26%; HC: 62%	MRI	Changes in cognition, depression, and anxiety symptoms in HF	Adults with HF experience increased symptoms of anxiety and depression
[26]	2014	heart failure	HF: 100	HF: 69.49	HF: 31%	transcranial Doppler ultrasonagraphy	Global cerebral blood flow velocity	Cerebral perfusion decline was associated with greater depressive symptoms
[27]	2014	heart failure	HF: 11; HC: 53	HF: 51.60; HC: 46.81	HF: 36%; HC: 40%	MRI spectroscopy	Assess anterior insular metabolites in HF	Significantly increased Cho/Cr ratios and reduced NAA/Cr levels, suggesting neuronal loss/dysfunction.
[28]	2009	heart failure	HF: 13; HC: 49	HF: 54.6; HC: 50.6	HF: 31%; HC: 41%	MRI	Extent of injury across the entire brain in HF	Brain structural injury emerged in areas involved in autonomic, pain, mood, language, and cognitive function
[29]	2013	heart failure	HF: 17; HC: 50	HF: 54.4; HC: 50.6	HF: 29%; HC: 42%	MRI	Mammillary bodies, frontal cortex, and hippocampi changes	Visual examination of brain MRI can detect damage in HF
[30]	2015	heart failure	HF: 931	HF: 75.9	HF: 52.3%	MRI	Association between cardiac hemodynamics and features of brain aging	Decrease in cardiac functioning is associated with brain alteration
[31]	2013	heart failure	HF: 69	HF: 68.07	HF: 42%	MRI	Associations between cognitive performance, physical fitness, and three indices of global brain integrity	Poor physical fitness is common in HF and associated with reduced structural brain integrity
